# Immunometabolic remodeling: new perspectives and strategies for liver transplantation

**DOI:** 10.3389/fimmu.2026.1776694

**Published:** 2026-04-02

**Authors:** Longbo Wang, Xiyang Sheng, Gengyuan Shi, Yongzhao Li, Dongdong Wang, Wei Wang, Chen Mi, Siyang Wang, Yongyue Du, Hanteng Yang

**Affiliations:** Department of General Surgery, Lanzhou University Second Hospital, Lanzhou, Gansu, China

**Keywords:** immune tolerance, immunometabolism, ischemia-reperfusion injury, liver transplantation, metabolic remodeling, post-transplant metabolic syndrome

## Abstract

Liver transplantation (LT) has become the optimal therapeutic strategy for end-stage liver disease. Beyond chronic conditions, acute liver failure (ALF) and the emerging field of transplant oncology have also become critical indications for LT. It is important to note that the systemic and local immunometabolic states in these specific pathologies may differ significantly from those in traditional end-stage liver disease, presenting unique challenges for immune management. With advancements in surgical techniques and perioperative management, the long-term survival rates of patients have significantly improved. However, extended patient survival and an expanding donor pool have unmasked long-term complications such as post-transplant metabolic syndrome (PTMS). Furthermore, the patient’s systemic metabolic state influences both the metabolism of immune cells and the utilization of immunosuppressants, posing severe challenges to patient management. Studies indicate that following liver transplantation, distinct immune cells undergo dynamic adaptive changes in energy metabolism, which directly determine the outcomes of rejection, ischemia-reperfusion injury (IRI), and immune tolerance. This review systematically elucidates the mechanisms of immune cell metabolic remodeling. Furthermore, it explores the translational prospects of targeting immunometabolic pathways to optimize immunosuppressive regimens, mitigating IRI, and establish non-invasive biomarkers for immune monitoring, ultimately providing new insights for improving the long-term outcomes of liver transplant recipients.

## 1 Introduction

The liver serves as the central hub of human nutritional metabolism and is also a vital immune organ, maintaining an intricate relationship with the overall metabolic status (1). As an effective treatment for end-stage liver disease, liver transplantation (LT) has significantly prolonged patient survival; however, postoperative management continues to face numerous challenges. First, to prevent graft rejection, patients require long-term administration of immunosuppressive drugs, such as calcineurin inhibitors (CNIs) and glucocorticoids. While suppressing the immune system, these drugs severely disrupt the body’s metabolic balance, leading to post-transplant metabolic syndrome (PTMS), which encompasses a spectrum of complications including new-onset diabetes, hypertension, hyperlipidemia, and obesity. Conversely, systemic metabolic abnormalities affect the efficacy of immunosuppressive agents and the metabolism of immune cells, presenting further therapeutic challenges. Second, the inevitable ischemia-reperfusion injury (IRI) during the perioperative period of liver transplantation triggers severe metabolic disturbances and inflammatory responses, compromising graft function. Finally, the process of establishing immune balance relies on traditional monitoring indicators that have limitations and fail to provide precise assessments.

Crucially, emerging research demonstrates that complex metabolic remodeling occurs as the organism adapts to the allograft and establishes immune tolerance. This process involves not only hepatic parenchymal cells but, more centrally, the profound metabolic reprogramming of various immune cell subsets (2). A deeper understanding of the mechanisms underlying this immunometabolic remodeling holds promise for providing novel perspectives to overcome the limitations of traditional immunosuppressants, develop targeted metabolic therapies, and optimize clinical management (3).

## 2 Metabolic dependency of immune cell function

**Figure 1:**
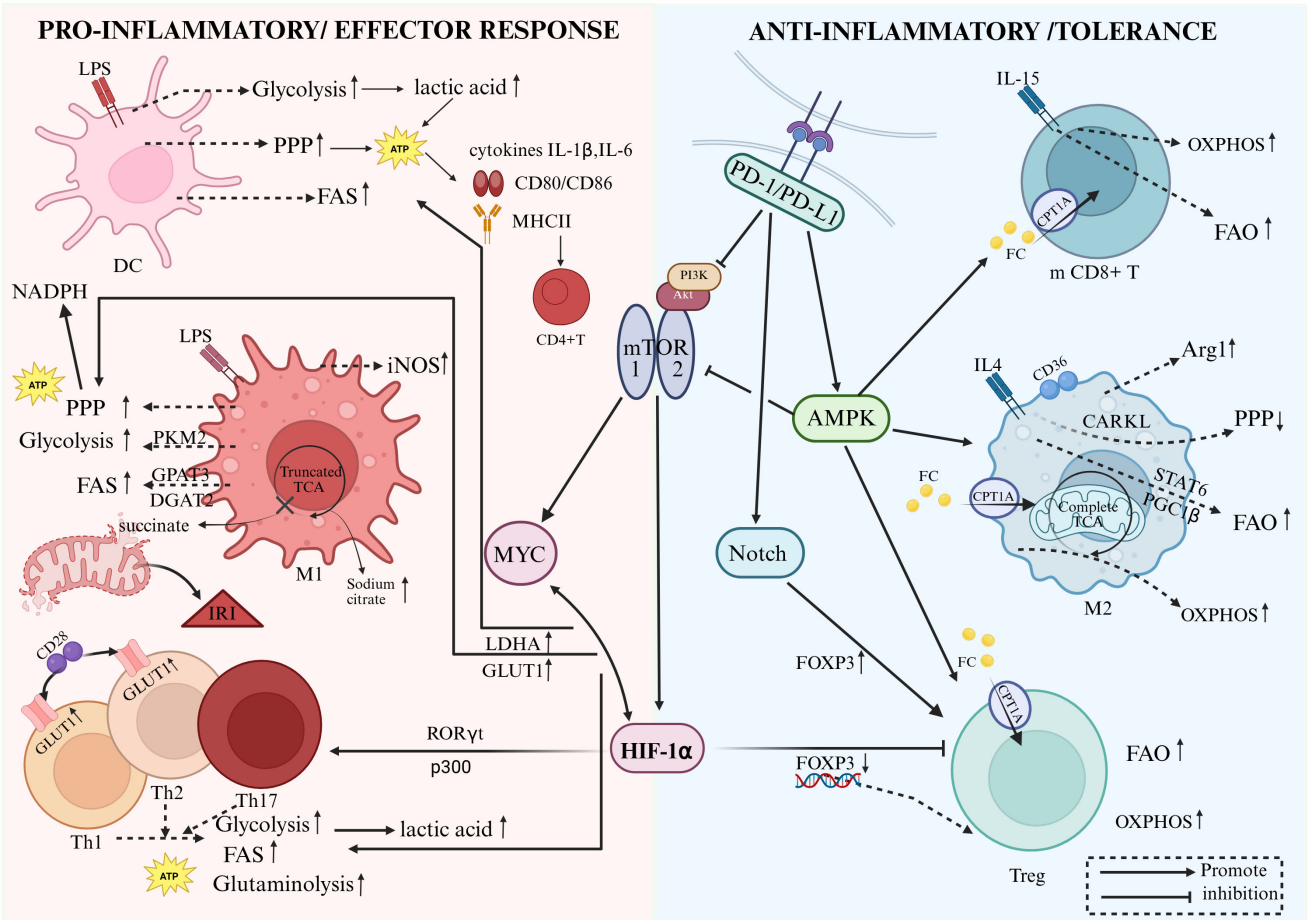
Metabolic reprogramming orchestrates immune cell differentiation and functional plasticity. The schematic illustrates the divergent metabolic programs characterizing pro-inflammatory effector cells (Left Panel) versus anti-inflammatory regulatory cells (Right Panel). (Left) Upon activation by pathogen-associated molecular patterns (e.g., LPS), Dendritic Cells (DCs) and M1 Macrophages undergo a “glycolytic switch” (Warburg effect). This process involves upregulation of the Pentose Phosphate Pathway (PPP) to generate NADPH and ATP, supporting the synthesis of pro-inflammatory cytokines (IL-1β, IL-6) and costimulatory molecules (CD80/CD86). A characteristic “truncated” TCA cycle in M1 macrophages leads to the accumulation of citrate (fueling Fatty Acid Synthesis, FAS, and NO production) and succinate. Succinate accumulation stabilizes HIF-1α, further driving glycolytic gene expression. Effector T cell subsets (Th1, Th2, Th17) similarly rely on aerobic glycolysis, glutaminolysis, and FAS, processes driven by the PI3K-Akt-mTOR-Myc signaling axis. (Right) In contrast, cells responsible for immune tolerance and resolution (M2 Macrophages, Tregs, and Memory CD8+ T cells) exhibit a metabolic preference for Oxidative Phosphorylation (OXPHOS) and Fatty Acid Oxidation (FAO). This metabolic profile is sustained by AMPK activation, which antagonizes mTOR signaling and promotes mitochondrial biogenesis via PGC-1α/β and STAT6 pathways. The PD-1/PD-L1 immune checkpoint reinforces tolerance by inhibiting glycolysis and promoting lipolysis and FAO. DC, Dendritic cell; LPS, Lipopolysaccharide; PPP, Pentose phosphate pathway; FAS, Fatty acid synthesis; FAO, Fatty acid oxidation; OXPHOS, Oxidative phosphorylation; TCA, Tricarboxylic acid cycle; HIF-1α, Hypoxia-inducible factor-1α; mTOR, Mammalian target of rapamycin; AMPK, AMP-activated protein kinase; PKM2, Pyruvate kinase M2; iNOS, Inducible nitric oxide synthase; Arg1, Arginase-1; CPT1A, Carnitine palmitoyltransferase 1A; RORγt, RAR-related orphan receptor γt.

Extracellular and intracellular signals regulate the activity of metabolic pathways, coupling cell growth and survival demands with metabolic mechanisms that generate key products to meet these needs. Similarly, in the context of immunity, specific alterations in metabolic pathways are integrated with immune effector functions. Immune cells undergo rapid clonal expansion and differentiation in response to activation signals. They swiftly remodel their metabolism to meet the bioenergetic and biosynthetic demands of rapid proliferation and to execute specific cellular functions (2). Generally, pro-inflammatory/effector cells tend to utilize glucose consumption (glycolysis) (4) and fatty acid synthesis (FAS) to support rapid proliferation, whereas anti-inflammatory/regulatory cells favor fatty acid oxidation (FAO) to maintain long-term survival (5) **Figure 1**. In the context of liver transplantation, understanding these distinct metabolic dependencies across different immune cell subsets (such as macrophages, DCs, and T cells) provides the essential theoretical foundation for targeted immunometabolic therapies.

### 2.1 Dendritic cell

As primary antigen-presenting cells (APCs), DCs are broadly defined by their function to capture, process, and present antigens to adaptive immune cells, thereby activating acquired immune responses (6). In the resting state, DCs maintain low energy consumption through oxidative phosphorylation (OXPHOS), focusing on antigen uptake. Upon detection of pathogens (e.g., TLR recognition of LPS), metabolism is immediately reprogrammed to glycolysis to provide energy for subsequent steps (7). The burst of glycolysis following activation produces significant amounts of lactate, which not only helps maintain an acidic microenvironment but also directly regulates the oligomerization of CCR7 (chemokine receptor), driving DC migration to secondary lymphoid organs (such as lymph nodes). ATP and NADH generated by glycolysis provide energy and substrates for the expression of inflammatory cytokines (e.g., IL-1β, IL-6) and costimulatory molecules (e.g., CD80/CD86), as well as for the synthesis of MHC class II molecule-peptide complexes, thereby activating T cells (8). Concurrently, DCs enhance the pentose phosphate pathway (PPP), generating NADPH to support lipid synthesis and endoplasmic reticulum expansion, which provides the necessary building blocks for DC activation and cytokine secretion (9). Interestingly, fatty acid synthesis is found to be upregulated during Toll-like receptor (TLR)-mediated DC activation. This increased fatty acid synthesis is closely associated with DC activation and the stimulation of CD8+ T cell responses, and it is crucial for maintaining specific membrane structures and signal transduction (9) (**Box 1**).

Box 1 **Key take-home messages - dendritic cells (DCs)**

• Core Mechanism: Activation (e.g., via TLRs) triggers a rapid “glycolytic switch” essential for DC migration and cytokine secretion.

• Evidence Level: High in Rodents (Murine BMDCs); Moderate in Humans (Human DCs show heterogeneity across subsets).

• Clinical Feasibility: Low. Specific targeting of DC metabolism *in vivo* is currently challenging due to off-target effects.

• Major Risks: Broad inhibition of glycolysis may impair antigen presentation, leading to infection susceptibility or vaccination failure

### 2.2 T cells

In the context of liver transplantation, the balance between allograft rejection and immune tolerance is largely dictated by how different types of T cells rely on distinct metabolic pathways during activation to mediate their immune responses (10, 11). Th1 cells participate in cell-mediated immunity, Th2 cells in humoral immunity, Th17 cells in mucosal immunity and inflammation, while Tregs are involved in immunosuppressive responses (12). Th1 and Th2 cells are highly dependent on glycolysis and glutaminolysis to support energetic and anabolic programs (13). In contrast, observations of naturally occurring and inducible FOXP3+ Tregs show that they rely on fatty acid uptake and β-oxidation to provide the necessary carbon, energy, and reducing equivalents for proliferation and regulatory functions (14). Th17 cells appear to be more plastic in their preferred metabolic state, relying on elements of glycolysis, oxidative phosphorylation, and β-oxidation to maintain their effector functions. The balance between Th17 and Treg cells is determined by HIF-1α, which promotes Th17 development via RORγt and p300, while simultaneously retarding Treg development by targeting FOXP3 for proteasomal degradation (2, 15).

Effector T cell subsets all exhibit increased glycolysis upon activation, most notably in Th17, Th1, and Th2 cells, as well as activated effector T cells. This is closely related to increased activity of the mTOR pathway associated with enhanced glycolysis and its downstream effector HIF-1α. Inhibition of the glycolytic process using 2-deoxyglucose (2-DG) promotes the conversion of Th17 cells into Treg cells (16).

Fatty acid oxidation (FAO) also plays a critical role in the generation and maintenance of Treg cells and memory CD8+ T cells (14). Tregs exhibit higher levels of FAO compared to Th1, Th2, and Th17 cells; furthermore, FAO promotes Treg generation while inhibiting effector T cell polarization (17). Memory CD8+ T cells proliferate slowly under homeostatic conditions and require FAO to respond promptly to antigenic stimulation. Stimulation of memory CD8+ T cells with IL-15 increases the expression of carnitine palmitoyltransferase 1a (CPT1A) and promotes fatty acid oxidation, thereby enhancing cell survival (18). Studies have shown that ligation of the inhibitory Programmed Death-1 (PD-1) receptor on T cells leads to increased CPT1A expression and elevated fatty acid oxidation (19). Furthermore, indirect activation of AMP-activated protein kinase (AMPK)—a cellular energy sensor and controller of FAO—using metformin increases the generation of CD8 memory T cells (20). In CD4+ T cell subsets, pharmacological or genetic inhibition of ACC1 indicates that fatty acid synthesis (FAS) is essential for the normal differentiation of Th17 cells, whereas it is not required for the production and function of Treg cells (21).

This differential expression is equally significant in T cell energy uptake; effector T cells upregulate the glucose transporter GLUT1, a process partially dependent on signaling from the costimulatory molecule CD28 (22). Failure of T cells to sufficiently enhance glucose metabolism results in reduced proliferation and cytokine production, while transgenic overexpression of GLUT1 receptors increases cytokine production and improves effector T cell survival (22). Additionally, naïve CD4+ T cells have an urgent requirement for glutamine to differentiate into Th1 and Th17 effector T cells, rather than Tregs. Restricting glutamine in the culture medium reduces proliferation and cytokine production in mitogen-stimulated lymphocytes (23) (**Box 2**).

Box 2 **Key take-home messages - T cells**

• Core Mechanism: Effector T cells (Th1/Th17) rely on mTOR-driven glycolysis; Tregs utilize AMPK-driven Fatty Acid Oxidation (FAO).

• Evidence Level: High in Humans. Validated by the clinical efficacy of CNIs and mTOR inhibitors.

• Clinical Feasibility: High. Existing drugs (Tacrolimus, Sirolimus) already target these pathways.

• Major Risks: Metabolic Syndrome. Systemic inhibition (e.g., by CNIs) causes hyperglycemia, dyslipidemia, and nephrotoxicity.

### 2.3 Macrophages

The differentiation processes of distinct macrophage subtypes depend on different metabolic pathways (24–26). M1 macrophages (induced by IFNγ and LPS stimulation) utilize glycolytic metabolism to promote inflammatory signaling (27), whereas M2 macrophages (defined as IL-4 activated) rely on a fatty acid oxidation program, which is promoted by Signal Transducer and Activator of Transcription 6 (STAT6) and PPARγ coactivatorβ (PGC1β), and inhibit inflammatory signaling (28). In the early stages of liver transplant ischemia-reperfusion injury, hepatic Kupffer cells predominantly polarize towards the M1 phenotype, relying on glycolysis to exacerbate inflammation; conversely, during the repair phase, a transition to the FAO-dependent M2 phenotype is required to promote tissue regeneration.

LPS can activate pyruvate kinase isozyme M2 (PKM2) to induce enhanced glycolysis in M1 macrophages. Moreover, PKM2 can translocate to the nucleus and interact with HIF-1α, promoting the expression of HIF-1α dependent genes, including those encoding glycolysis-related enzymes and inflammatory cytokines such as IL-1β (29). When PKM2 enters a tetrameric state and is unable to enter the nucleus, it shifts the macrophage gene expression profile towards M2, thereby reducing LPS-induced glycolysis.

The pentose phosphate pathway (PPP) has been shown to be upregulated in M1 macrophages (30). The PPP not only generates pentoses and ribose-5-phosphate for nucleic acid production but is also a major source of NADPH (31). In contrast, M2 macrophages appear to inhibit the PPP. Regulation of the PPP in macrophages is controlled by carbohydrate kinase-like protein (CARKL), a sedoheptulose kinase. CARKL is upregulated in response to IL-4 but inhibited in response to LPS, leading to PPP suppression in M2 macrophages (32). The downregulation of CARKL appears crucial for the metabolic shift of glucose from aerobic metabolism to glycolysis and for the PPP in M1 macrophages.

Macrophage subtypes also exhibit differences in the TCA cycle. M2 macrophages possess an intact TCA cycle coupled with oxidative phosphorylation (24, 30), a process dependent on glutamine-related metabolism and the UDP-GlcNAc pathway (30). However, in M1 macrophages, the TCA cycle has been proven to be truncated at two points—after citrate and after succinate (24, 33). Accumulated citrate in M1 macrophages is exported from the mitochondria via the citrate transporter. It is then utilized for fatty acid production, which in turn supports membrane biogenesis. This truncated TCA cycle is also observed in activated DCs and appears particularly important for their function, as these cells require extensive membrane production to support antigen presentation. Simultaneously, excess citrate can generate prostaglandins, key effector molecules produced by macrophages. Accumulated succinate promotes the production of the pro-inflammatory cytokine IL-1β via HIF-1α in activated macrophages, contributing to IRI in liver transplant donors (34).

Fatty acid synthesis (FAS) is closely associated with the pro-inflammatory effector functions of M1 macrophages (25). In LPS-activated macrophages, carbon derived from glucose via increased glycolysis rates preferentially enters the fatty acid pool (35). This manifests as increased accumulation of triglycerides and cholesterol esters. This accumulation is largely due to increased *de novo* fatty acid synthesis and elevated expression of multiple key enzymes involved in glycerolipid synthesis [including glycerol-3-phosphate acyltransferase (GPAT3), Lipin-1, and diacylglycerol O-acyltransferase 2 (DGAT2)] (25, 35). M2 macrophages rely on FAO, a metabolic switch mediated primarily by STAT6 and PGC1β (28, 36), and associated with increased expression of CPT-1A, CD36, and Medium-Chain Acyl-CoA Dehydrogenase (MCAD) (36). Promoting fatty acid oxidation by overexpressing CPT-1A in human adipose tissue macrophages can reduce inflammatory responses, improve adipocyte insulin sensitivity, lower endoplasmic reticulum stress, and decrease reactive oxygen species damage in macrophages (37).

Amino acid metabolism is also inextricably linked to macrophages; differences in arginine metabolism remain one of the most reliable factors for distinguishing M1 and M2 macrophages (38). Depending on the activation type, enzymes responsible for arginine metabolism are induced in macrophages. In murine M1 macrophages, inducible nitric oxide synthase (iNOS) is upregulated, leading to the breakdown of arginine into citrulline and nitric oxide, the latter playing a key role in intracellular pathogen killing whereas in humans, the role of iNOS remains less distinct (38, 39). Conversely, in M2 macrophages, Arginase-1 (Arg1) is induced, producing urea, polyamines, and ornithine, which are critical for the wound-healing functions of this macrophage population (38) (**Box 3**).

Box 3 **Key take-home messages – macrophages**

• Core Mechanism: M1 polarization requires a “truncated TCA” cycle and glycolysis; M2 relies on intact TCA and FAO.

• Evidence Level: High in Rodents; Variable in Humans. Notably, the iNOS/Arginine metabolism binary is distinct in mice but less defined in human macrophages.

• Clinical Feasibility: Moderate. Pharmacological modulation (e.g., GLP-1 analogs, 5-ALA) shows promise in ameliorating IRI-induced M1 polarization in rodent models.

• Major Risks: Inhibiting M1 polarization too early may hinder pathogen clearance during the perioperative period.

### 2.4 Synergistic regulatory mechanisms of immunometabolic networks

Metabolic alterations in immune cells are systemic processes driven collectively by multiple signaling pathways. This metabolic reprogramming does not occur in isolation but forms a tight interaction network with cytokine signaling, canonical metabolic sensing pathways, and metabolites. Understanding and precisely regulating these networks provides an important theoretical and practical basis for post-liver transplant immunotherapy. Among these, molecules such as MYC, HIF-1α, AMPK, mTOR, and PD-1/PD-L1 have demonstrated potential in regulating immune cell function (40).

MYC: A core driver of cell proliferation and metabolism. It synergizes with HIF-1α to upregulate glucose transporter (GLUT1), lactate dehydrogenase A (LDHA) which catalyzes the conversion of pyruvate to lactate, and molecules related to glutamine metabolism. This enhances anabolic pathways such as glycolysis and glutaminolysis to meet the biomass demands of rapidly proliferating effector T cells, serving as a central driver of immune cell metabolic activation (41).

Hypoxia-Inducible Factor (HIF-1α): Stably expressed during hypoxia or mTOR activation. It promotes the expression of glycolysis-related genes (e.g., GLUT1, HK2, LDHA) while inhibiting oxidative phosphorylation, enabling cells to adapt to hypoxic environments and rapidly generate energy. HIF-1α interacts with Myc to collectively amplify the glycolytic program (42). In Th17 cell differentiation, it synergizes with RORγt to activate pro-inflammatory genes while inhibiting Treg cell development, thereby regulating immune balance (15). Additionally, in liver transplant reperfusion injury, HIF-1α can drive cellular pro-inflammatory activity, exacerbating tissue damage (43).

Mammalian Target of Rapamycin (mTOR): Regulates metabolism and immune cell differentiation via mTORC1/2 complexes: mTORC1 promotes glycolysis and protein synthesis, supporting the functions of effector T cells such as Th1 and Th17; mTORC2 drives Th2 cell differentiation by regulating mitochondrial metabolism (44). Inhibition of mTOR (e.g., via rapamycin) can induce a metabolic shift in T cells towards OXPHOS and promote Treg generation, serving as a clinically common target for inducing immune tolerance (45). Furthermore, mTORC1 can upregulate the expression of Myc and HIF-1α, thereby driving the differentiation and function of effector T cells.

AMP-Activated Protein Kinase (AMPK): As a cellular energy sensor, it acts alongside mTOR as a key antagonistic pair in sensing cellular energy status and regulating metabolism. Upon activation, it inhibits lipid synthesis and suppresses mTORC1 activity (46), thereby promoting oxidative phosphorylation (OXPHOS) and fatty acid oxidation to maintain cellular energy homeostasis (47). In liver transplantation, AMPK improves hepatocyte energy status, attenuates oxidative stress and IRI, and regulates immune tolerance by inhibiting effector T cell glycolysis and enhancing Treg cell FAO, thereby reducing rejection risk (48).

PD-1/PD-L1 Pathway: Upon binding, it blocks the PI3K/Akt/mTOR and Ras/MAPK/ERK pathways, inhibiting effector T cell glycolysis and activation (49). Simultaneously, it induces Treg proliferation and stabilizes Foxp3 expression via the Notch pathway, creating an immunosuppressive microenvironment that plays a critical role in transplant immune tolerance (50).

These molecules constitute a dynamically balanced regulatory network. Under conditions of antigen stimulation and nutrient sufficiency, the PI3K/Akt/mTOR pathway is activated, driving Myc and HIF-1α expression to promote glycolysis and biosynthesis, thereby supporting the differentiation and function of effector T cells (e.g., Th1, Th17). Under nutrient deprivation or PD-1 signal activation, AMPK activity is enhanced, inhibiting mTOR and subsequently downregulating Myc and HIF-1α. This promotes oxidative metabolism, favoring cell subsets with long-term survival and suppressive functions, such as memory T cells and Tregs.

## 3 Clinical challenges: the interplay between immunosuppressants and metabolic disorders

Traditional immunosuppressants (glucocorticoids, calcineurin inhibitors such as tacrolimus, mTOR inhibitors, etc.) achieve effective T cell suppression by blocking the activation of NFAT, NF-κB, and AP-1. However, this broad T cell suppression can induce Post-Transplant Metabolic Syndrome (PTMS) (51). More importantly, the host’s baseline metabolic status (e.g., fatty liver, diabetes, obesity) can inversely weaken the efficacy of immunosuppressants and interfere with the establishment of immune tolerance by altering CYP3A4 enzyme activity or inducing chronic low-grade inflammation, constituting a dual challenge for clinical management.

### 3.1 Calcineurin inhibitors

CNIs are currently widely used agents for preventing acute rejection and maintaining immune balance following liver transplantation. They primarily function by binding to the immunophilin FKBP12 in the T cell cytoplasm to form a complex, which inhibits the phosphatase activity of calcineurin, thereby blocking the Ca²^+^/calmodulin signaling pathway. Studies report that tacrolimus blocks the NFAT signaling pathway, preventing its dephosphorylation and nuclear translocation. This subsequently inhibits the transcription of key cytokines for T cell activation, such as IL-2, IL-4, and IFN-γ, arresting T cells in the G_0_/G_1_ phase and indirectly inhibiting B cell differentiation and antibody production (52).

#### 3.1.1 Tacrolimus

Although tacrolimus has been the mainstay of anti-rejection therapy in liver transplantation for the past decade, its adverse metabolic effects are garnering increasing attention. Tacrolimus impairs insulin production and secretion in pancreatic β-cells and induces apoptosis, thereby reducing β-cell survival. The primary mechanism involves interference with the Glucagon-Like Peptide-1 (GLP-1)-mediated pathway, where CREB and NFAT normally stimulate Insulin Receptor Substrate 2 (IRS2) to increase insulin secretion. Furthermore, tacrolimus inhibits glucose uptake in adipocytes and muscle cells in a dose-dependent manner, potentially by reducing the levels of Glucose Transporter 4 (GLUT-4) on the cell membrane via increased endocytosis, ultimately leading to Post-Transplant Diabetes Mellitus (PTDM) (52). Beyond its impact on glucose metabolism, CNI-induced vascular endothelial injury and arteriolar vasoconstriction can lead to graft ischemia and chronic renal hypoperfusion, as well as hypertension and electrolyte disturbances (53).

#### 3.1.2 Cyclosporine A

Compared to tacrolimus, cyclosporine causes more severe interference with lipid metabolism, reducing the clearance of circulating low-density lipoprotein (LDL) cholesterol. Potential mechanisms may involve reduced transport of cholesterol into bile and the intestine, decreased bile acid synthesis, and interference with LDL receptor binding (54).

### 3.2 Corticosteroids

Corticosteroids, possessing broad anti-inflammatory and immunosuppressive properties, are among the most frequently used immunosuppressants following liver transplantation. Agents such as prednisone and methylprednisolone function by inhibiting pro-inflammatory cytokine production, reducing antigen-presenting cell activation, and suppressing effector T cell activity. Corticosteroids are typically administered early after transplantation to prevent acute rejection and control inflammation. However, long-term use leads to various adverse effects, including hyperglycemia, hypertension, osteoporosis, and increased risk of infection (55). Consequently, many transplant centers have adopted steroid-sparing or withdrawal protocols aimed at minimizing or discontinuing their use in patients with stable graft function.

### 3.3 Nucleotide metabolism inhibitors

Mycophenolate Mofetil (MMF), the 2-morpholinoethyl ester derivative of mycophenolic acid, is widely used as an antiproliferative immunosuppressant following liver transplantation. MMF is hydrolyzed *in vivo* to its active metabolite, mycophenolic acid (MPA). MPA reversibly inhibits inosine monophosphate dehydrogenase (IMPDH), thereby blocking the *de novo* synthesis of guanosine monophosphate (GMP). Since lymphocytes lack key enzymes for the purine salvage pathway and are highly dependent on the *de novo* synthesis pathway for proliferation, MPA specifically inhibits the proliferation of T and B lymphocytes (56). While long-term MPA use may cause bone marrow suppression and gastrointestinal reactions, it has minimal inherent nephrotoxicity and a relatively mild direct impact on blood glucose and lipids. Therefore, it is considered to have a relatively favorable metabolic profile for recipients at high risk of metabolic syndrome and is often used to spare or replace CNIs. The “triple regimen” combining MMF with a CNI (cyclosporine/tacrolimus) and glucocorticoids is currently one of the standard maintenance immunosuppressive protocols in liver transplantation (57).

### 3.4 mTOR pathway inhibitors

mTOR inhibitors, including sirolimus and everolimus, have become valuable alternatives to CNIs in liver transplantation due to their ability to selectively inhibit effector T cell proliferation while relatively preserving the Treg cell. Additionally, mTOR inhibitors possess anti-proliferative effects that can significantly reduce the risk of recurrence in patients transplanted for hepatocellular carcinoma (HCC), making them particularly suitable for recipients with malignancies (58).

Despite these advantages, significant side effects exist (59). Common adverse reactions include hyperlipidemia and New-Onset Diabetes Mellitus (NODM). mTOR inhibition reduces mitochondrial ATP production in β-cells, impairing their function and decreasing insulin secretion, which hinders β-cell adaptation to hyperglycemia and exacerbates the diabetic state. Moreover, mTOR inhibition reduces the catabolism of circulating lipoproteins by inhibiting lipase activity, leading to dyslipidemia (60). This “double-edged sword” effect on metabolic regulation (inhibiting immune cell glycolysis while inducing systemic hyperlipidemia) necessitates rigorous patient selection and risk assessment in clinical practice.

### 3.5 Impact of metabolic diseases on immunosuppression and immune cells

The liver is the primary site for the metabolism of CNIs (e.g., tacrolimus, cyclosporine) and mTOR inhibitors. Studies indicate that Metabolic dysfunction-associated steatotic liver disease (MASLD) significantly alters the activity of hepatic drug-metabolizing enzymes, thereby affecting the blood concentrations of immunosuppressants. For instance, Cytochrome P450 3A4 (CYP3A4) is a key enzyme for the metabolism of tacrolimus and cyclosporine (61). Multiple studies confirm that in patients with fatty liver (especially MASH accompanied by inflammation), the protein expression and enzymatic activity of CYP3A4 are significantly decreased (potentially by 30%-60%). This leads to potential drug accumulation in recipients with fatty liver under the same dosage, increasing the risks of nephrotoxicity and neurotoxicity (62). Additionally, as most immunosuppressants are lipophilic, excessive adipose tissue in obese or hyperlipidemic patients can lead to drug sequestration, increasing the apparent volume of distribution. This results in failure to achieve steady-state concentrations or delayed drug release during tapering (63), thereby complicating therapeutic management.

Research indicates that hyperglycemia exacerbates rejection risk by fueling the mTOR-HIF-1α axis. This signaling cascade, which fundamentally drives Th1/Th17 differentiation (detailed in Section 2.4), is hyper-activated by high glucose availability, thereby shifting the immune balance away from tolerogenic Tregs. This metabolic bias predisposes immune cells in a hyperglycemic environment to be inherently more pro-inflammatory and prone to rejection.

In obese and fatty liver hosts, excessive circulating free fatty acids (FFAs) and adipokines (e.g., leptin) induce tissue-resident macrophages to polarize towards the pro-inflammatory M1 phenotype (64). This persistent chronic low-grade inflammation (“Meta-inflammation”) not only disrupts the liver’s tolerogenic microenvironment but may also further weaken Treg suppressive function through the secretion of IL-6 and TNF-α (65).

## 4 New therapeutic strategies targeting immunometabolism

Establishing immune balance after liver transplantation is crucial. Emerging evidence strongly suggests that metabolic remodeling plays a crucial role in immune cell activation, differentiation, and the regulation of immune rejection. By investigating molecular mechanisms and targeting immune-related metabolic signal transduction and metabolites to shape immune responses, we can provide new targets and therapeutic options for balancing immune tolerance and reducing the incidence of metabolic syndrome caused by long-term immunosuppressant use (10).

### 4.1 Optimizing clinical immunosuppression regimens

To address immunosuppressant-induced metabolic syndrome, individualized adjustment of immunosuppressant usage based on specific clinical manifestations and combination therapies should be employed to avoid damage caused by excessive single-agent dosages (66).

Reducing CNI exposure can lower the risk of post-transplant NODM. A retrospective study of 973 transplant recipients showed that the mean concentration of tacrolimus (cTAC) in the NODM group was significantly higher than in the non-NODM group. Furthermore, recipients who maintained a lower time-weighted average tacrolimus concentration (<5.89 ng/mL within that specific cohort) exhibited a significantly reduced incidence of obesity, dyslipidemia, chronic renal insufficiency, and moderate-to-severe infection compared to the high-concentration group (67). However, because optimal target trough concentrations vary widely across transplant centers and patient profiles, this specific value should be viewed as an illustrative benchmark for CNI minimization rather than a universal clinical guideline. In practice, treatment must be strictly tailored; for example, recipients with preexisting fatty liver or obesity require vigilant therapeutic drug monitoring and often significantly lower starting doses of tacrolimus or cyclosporine. For those with renal impairment, a promising CNI-sparing strategy is combining low-dose CNIs with mTOR inhibitors (e.g., sirolimus or everolimus). This combination helps preserve the Treg population and maintain its suppressive function, potentially reducing rejection rates (68). Mortality, acute rejection rates, and graft loss rates are similar between liver transplant recipients on everolimus and those on CNIs. Everolimus can effectively and safely prevent post-transplant renal impairment when used to replace or reduce CNIs, or when combined with MMF (57). A prospective, multi-center study found that everolimus enables early tacrolimus minimization, thereby improving renal function (69). Moreover, this combination therapy is particularly beneficial for patients with a history of HCC (70). However, given that mTOR inhibitors themselves can cause metabolic side effects such as hyperlipidemia, clinicians must weigh the pros and cons when adopting CNI minimization protocols combined with mTOR inhibitors (59).

#### 4.1.1 Immunosuppression withdrawal and treg-targeted therapeutic strategies

Immunosuppression withdrawal attempts can be successful in a selected minority of long-term surviving adult and pediatric LT recipients (71–73). Although attempts at complete discontinuation of immunosuppression result in a high incidence of allograft rejection, rejection episodes are often mild and responsive to transient increases in immunosuppression. However, the safety of such protocols when conducted under very close biochemical and histological monitoring requires further investigation.

Unlike traditional immunosuppressive therapies that nonspecifically suppress immune responses, Tregs offer a targeted immunomodulatory approach, selectively suppressing alloimmune responses while preserving overall immune function. This unique ability to modulate alloreactivity without compromising systemic immunity makes Tregs a promising target for therapeutic strategies aiming to induce transplant tolerance (68, 74). Emerging therapies focused on preserving or enhancing Treg function include mTOR inhibitors that favor Treg metabolic advantages, low-dose IL-2 therapy, and Treg-based cell therapies. Clinical trial results using cell therapies enriched with ex vivo-expanded regulatory T cells have shown safety and efficacy in drug reduction and tolerance induction (75), though these results need confirmation in further trials.

#### 4.1.2 Metabolic support therapy from an immunological perspective (diet/exercise/bariatric surgery)

Beyond direct pharmacological intervention, improving the patient’s systemic metabolic state is a crucial adjunctive measure for regulating the immunometabolic microenvironment. Obesity and hyperlipidemia resulting from PTMS are not only cardiovascular risk factors but also catalysts for a pro-inflammatory immune state. Dietary control and regular exercise can not only reduce weight but may also alleviate pro-inflammatory metabolic programs. Studies show that bariatric surgery or strict dietary intervention can lower circulating free fatty acid levels, thereby restoring the metabolic advantage and suppressive function of Treg cells.

A single-center cross-sectional study analyzing the correlation between exercise/diet and metabolic syndrome in pediatric liver transplant patients suggested that muscle atrophy, weight gain, and reduced physical activity are positively correlated with the incidence of metabolic syndrome (76). A prospective study randomized patients into two groups: Exercise and Dietary intervention (ExD) and Usual Care (UC). After one year of observation, although the intervention group showed relative improvement in exercise capacity, no differences in body composition were detected between the groups (77).

A multicenter retrospective cohort study analyzed patients receiving Liver Transplantation combined with Sleeve Gastrectomy (LTSG) under a single clinical protocol versus BMI >30 patients receiving LT alone for MASLD. At 8-year follow-up, compared to LT alone, patients receiving LTSG showed significantly lower prevalence of diabetes, hypertension, graft steatosis, and fibrosis, as well as better weight control (78).

#### 4.1.3 Balancing antitumor immunity and allograft rejection: the challenge of ICI bridging therapy

With the rise of transplant oncology, immune checkpoint inhibitors (ICIs), particularly PD-1/PD-L1 inhibitors, are increasingly utilized for hepatocellular carcinoma (HCC) downstaging to meet transplantation criteria (79). Mechanistically, ICIs restore anti-tumor immunity by reinvigorating glycolysis and mitochondrial function in exhausted T cells. However, this drug-induced metabolic hyperactivation poses a severe risk of acute rejection (AR) or even fatal hepatic necrosis if persisting at the time of transplantation. Consequently, a mandatory “washout period” is clinically advocated to allow systemic immunometabolism to normalize, illustrating the critical need to balance anti-tumor efficacy with graft safety (80).

Furthermore, post-transplant management faces a similar immunometabolic dilemma regarding tumor recurrence. Traditional CNIs, while effective for rejection, may impair immune surveillance and induce metabolic syndrome, creating a pro-tumorigenic microenvironment (81). Conversely, metabolic modulators or mTOR inhibitors may offer a dual advantage: maintaining graft tolerance via specific metabolic checkpoints (e.g., promoting fatty acid oxidation in Tregs) while preserving sufficient anti-tumor surveillance to prevent HCC recurrence.

### 4.2 Establishing immune tolerance by directly targeting metabolic pathways

As previously described, different immune cells rely on distinct metabolic pathways and signaling cascades to generate immune effects, creating cellular metabolic selectivity (17). This differential metabolic requirement allows for more precise mediation of immunosuppression, avoiding the metabolic syndrome caused by traditional immunosuppressants and reducing the risks of infection and tumor recurrence. Selectively suppressing immune responses via metabolic inhibitors (MIs), rather than the broad blockade seen with traditional agents, is highly attractive for liver transplant immunotherapy (82).

Given the absolute dependence of effector T cells on aerobic glycolysis for clonal expansion (detailed in Section 2.2), glycolytic inhibitors such as 2-deoxyglucose (2-DG), anti-type II diabetes drugs (metformin), and glutamine metabolism inhibitors (DON) have been shown to prevent or delay graft rejection in murine skin and heart allograft models (10, 83), as well as reduce viral infection and cancer recurrence (84, 85). However, the direct application of broad-spectrum metabolic inhibitors faces significant clinical challenges. Since glycolysis is also the basal metabolic mode for neurons, cardiomyocytes, and regenerating hepatocytes, systemic use of 2-DG may lead to severe central nervous system toxicity and delayed recovery of liver function.

**Figure 2:**
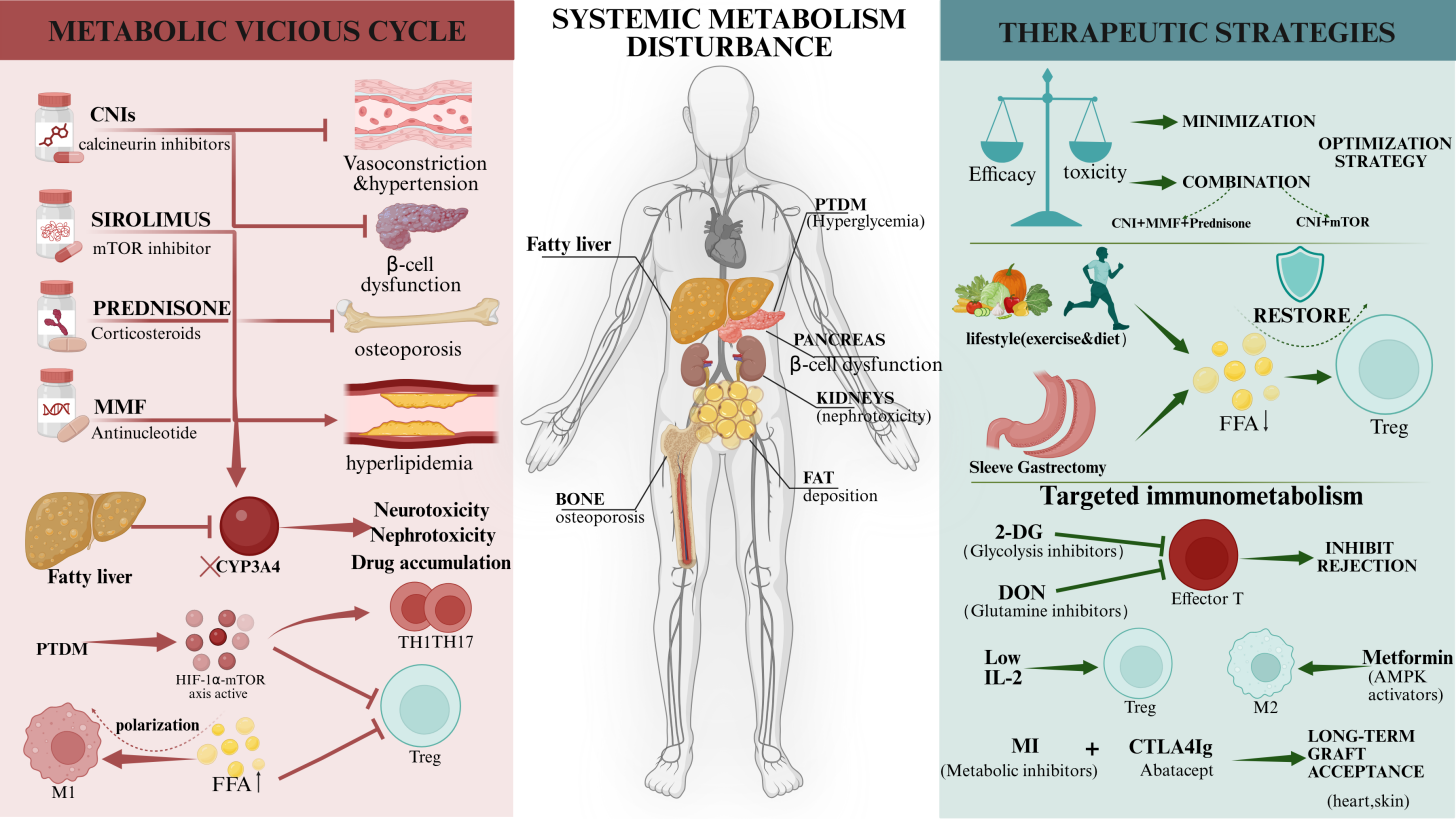
The interplay between immunosuppression-induced metabolic disorders and targeted therapeutic strategies. (Left Panel) The Metabolic Vicious Cycle: Standard immunosuppressive regimens (CNIs, mTOR inhibitors, Corticosteroids) are associated with systemic metabolic adverse events, including hypertension, β-cell dysfunction, osteoporosis, and dyslipidemia. Conversely, pre-existing host metabolic disorders (e.g., Fatty Liver/MASLD) downregulate hepatic CYP3A4 activity, leading to drug accumulation and enhanced toxicity. Systemic metabolic disturbances, such as elevated Free Fatty Acids (FFA) and hyperglycemia, promote a pro-inflammatory microenvironment by driving Th1/Th17 polarization via the mTOR/HIF-1α axis while suppressing Treg differentiation. (Right Panel) Therapeutic Strategies: Clinical management involves balancing efficacy and toxicity through minimization strategies or combination therapies (e.g., reduced CNI + mTOR inhibitors). Lifestyle interventions (Diet, Exercise) and Bariatric Surgery (Sleeve Gastrectomy) help restore metabolic homeostasis. Emerging “Targeted Immunometabolism” approaches aim to induce graft tolerance using specific metabolic modulators: 2-DG (Glycolysis inhibitor) and DON (Glutamine inhibitor) to suppress effector T cells; Metformin to activate AMPK and enhance M2/Treg function; and synergistic combinations with CTLA4-Ig to promote long-term graft acceptance. CNI, Calcineurin inhibitors; MMF, Mycophenolate mofetil; PTDM, Post-transplant diabetes mellitus; FFA, Free fatty acids; CYP3A4, Cytochrome P450 3A4; 2-DG, 2-Deoxy-D-glucose; DON, 6-Diazo-5-oxo-L-norleucine.

Combining traditional immunosuppressants with metabolic inhibitors is also being explored in transplantation research, such as combining CTLA4-Ig (Abatacept) with metabolic inhibitor therapy. Adding CTLA4-Ig to continuous metabolic therapy not only improves skin allograft survival but also promotes long-term acceptance of heart allografts without maintenance therapy (86). **Figure 2**.

### 4.3 Targeting metabolism-inflammation to alleviate IRI

Ischemia-Reperfusion Injury (IRI) is a common sterile inflammatory response following liver transplantation, characterized by the interaction between metabolic disturbances and immune activation. During the ischemic phase, oxygen deprivation leads to ATP depletion in hepatocytes, necessitating a shift to anaerobic glycolysis, which results in lactate accumulation and intracellular acidosis (87). During reperfusion, mitochondria generate excessive reactive oxygen species (ROS), triggering oxidative stress that causes extensive hepatocyte death and the release of damage-associated molecular patterns (DAMPs)—such as HMGB1, succinate, and nucleic acids. These DAMPs subsequently activate innate immune cells, including Kupffer cells and neutrophils (4, 88). Macrophages secrete pro-inflammatory cytokines (e.g., TNF-α, IL-1β, IL-6) to initiate the inflammatory cascade, while neutrophils exacerbate oxidative stress by releasing ROS via the “respiratory burst” (89). Concurrently, dendritic cells (DCs) upregulate MHC class II and costimulatory molecules, establishing conditions primed for T cell activation (88). Cytokines secreted by innate immune cells, such as IL-12, IL-18, and IFN-γ, drive the differentiation of naïve T cells toward Th1/Th17 phenotypes while suppressing Tregs, thereby fostering a pro-inflammatory microenvironment (90). Furthermore, complement activation products (e.g., C3b) assist B cells in producing natural IgM/IgG, amplifying antibody-mediated hepatocyte injury, including ferroptosis, apoptosis, necrosis, and pyroptosis (91, 92). Ultimately, a vicious cycle forms between hepatocyte injury and immune cell chemotaxis/activation, exacerbating the inflammatory response (**Box 4**).

Box 4 **Key Take-home messages - ischemia-reperfusion injury (IRI)**

• Core Mechanism: Ischemic accumulation of succinate drives mitochondrial ROS bursts (via RET) upon reperfusion.

• Evidence Level: High in Mice (Seminal metabolomic studies); Moderate in Humans (Lactate/Acidosis confirmed).

• Clinical Feasibility: Moderate to High. Strategies such as Metabolic Preconditioning (e.g., Fasting) and Inhaled NO have demonstrated therapeutic potential in reducing oxidative injury.

• Major Risks: Timing. Metabolic interventions must be precise; “washout periods” are critical when bridging with other therapies.

#### 4.3.1 Metabolic remodeling

Metabolic remodeling is intrinsic to the entire process of IRI. During ischemia, ATP depletion and enhanced anaerobic glycolysis lead to intracellular acidosis and ionic imbalance, causing mitochondrial dysfunction (87). Upon reperfusion, mitochondria generate massive amounts of ROS, triggering oxidative stress (93). During this period, TCA cycle intermediates such as succinate accumulate and trigger an ROS burst via reverse electron transport (RET), amplifying oxidative damage (94). This disrupts mitochondrial oxidative phosphorylation (OXPHOS) and the TCA cycle, impairs fatty acid β-oxidation, and exacerbates lipid accumulation and peroxidation, ultimately inducing ferroptosis (92).

#### 4.3.2 Therapeutic strategies

Progress has been made in targeting immune cell metabolism to ameliorate IRI (17, 95, 96). Inhibiting glycolytic pathways and associated metabolites can improve the acidic microenvironment, suppress immune cell activation, and attenuate hepatocyte injury. For instance, in mouse IRI models, hepatocyte HSPA12A attenuates IRI by inhibiting glycolysis-mediated lactylation and exosomal secretion of HMGB1, thereby blocking macrophage chemotaxis and inflammatory activation (97). Additionally, studies in mice demonstrate that fasting-associated Sirt1 activity downregulates circulating HMGB1 levels, inhibiting innate immune responses and exerting hepatoprotective effects (98). Pre-clinical evidence in murine models indicates that hepatocyte Insig2 protects against hepatic IRI by optimizing glucose metabolism via the pentose phosphate pathway (PPP) to enhance antioxidant defense and redox homeostasis (99). Similarly, in rodent experiments, liraglutide alleviates hepatocyte apoptosis by inhibiting IRI-induced M1 polarization in Kupffer cells via GLP-1 signaling (100).

#### 4.3.3 Dual role of HIF-1α

HIF-1α plays a dichotomous role during IRI. While its initial activation supports metabolic adaptation via glycolysis (as described in Section 2.4) (101). its sustained activity during severe hypoxia exacerbates tissue injury by driving excessive lactate accumulation and inflammatory cytokine release (102, 103). Therefore, hepatoprotection can be achieved by directly or indirectly inhibiting HIF-1α using small molecule inhibitors such as 2-DG, 3-BP, or PX-478. Furthermore, activating AMPK not only suppresses adhesion molecule expression and leukocyte infiltration but also promotes cell survival via anti-apoptotic mechanisms, attenuates oxidative stress, and maintains ATP levels (48, 104).

#### 4.3.4 Mitochondrial metabolic targets

Preserving mitochondrial function, promoting fatty acid β-oxidation (FAO), and reducing TCA intermediate accumulation are critical for alleviating IRI. Targeting the pathological accumulation of succinate is critical (95, 105). As detailed in Section 2.3, seminal metabolomic studies in mice identified that succinate accumulation (resulting from the ‘truncated’ TCA cycle) drives mitochondrial ROS bursts via reverse electron transport (RET) (94, 106). Consequently, pharmacological inhibition of ischemic succinate accumulation effectively alleviates IRI (94).

Mechanistic studies in murine models reveal that acetate accumulates significantly following hepatic ischemia-reperfusion injury (HIRI). Upon uptake by CD8+ T cells, it expands the acetyl-CoA pool, leading to the acetylation of the key glycolytic enzyme GAPDH, which subsequently enhances the proliferation and differentiation of CD8+ T cells (107, 108). Other metabolites, such as itaconate, also play pivotal roles. Generated by Acod1 (109), itaconate inhibits SDH-mediated succinate oxidation (110) and enhances antioxidant gene expression by activating the Nrf2 pathway, thereby reversing M1 macrophage-mediated injury (94, 95).

Enhancing FAO is similarly protective: mild hypothermia increases FAO by activating the JAK2/STAT3-CPT1a pathway in Kupffer cells, thereby mitigating inflammation (111). As a master regulator driving FAO and mitochondrial biogenesis, PGC-1α classically supports M2-like anti-inflammatory phenotypes to protect the liver. However, its immunomodulatory role is highly context-dependent; in specific pathological settings like IRI, PGC-1α can adaptively inhibit NF-κB signaling and excessive M2 polarization to alleviate hepatic fibrosis (112). Furthermore, PGC-1α enhances this protective effect by modulating intracellular ROS accumulation (113). Experimental data in mice suggests that 5-Aminolevulinic acid (5-ALA) accelerates lipid catabolism and suppresses excessive mitochondrial ROS generation, thereby attenuating hepatocyte injury. Furthermore, 5-ALA not only inhibits ATP production via dual pathways in M0 macrophages but also significantly reduces LPS-induced M1 polarization while enhancing IL-4-induced M2 polarization, ultimately fostering an anti-inflammatory microenvironment to facilitate tissue repair (114).

**Figure 3:**
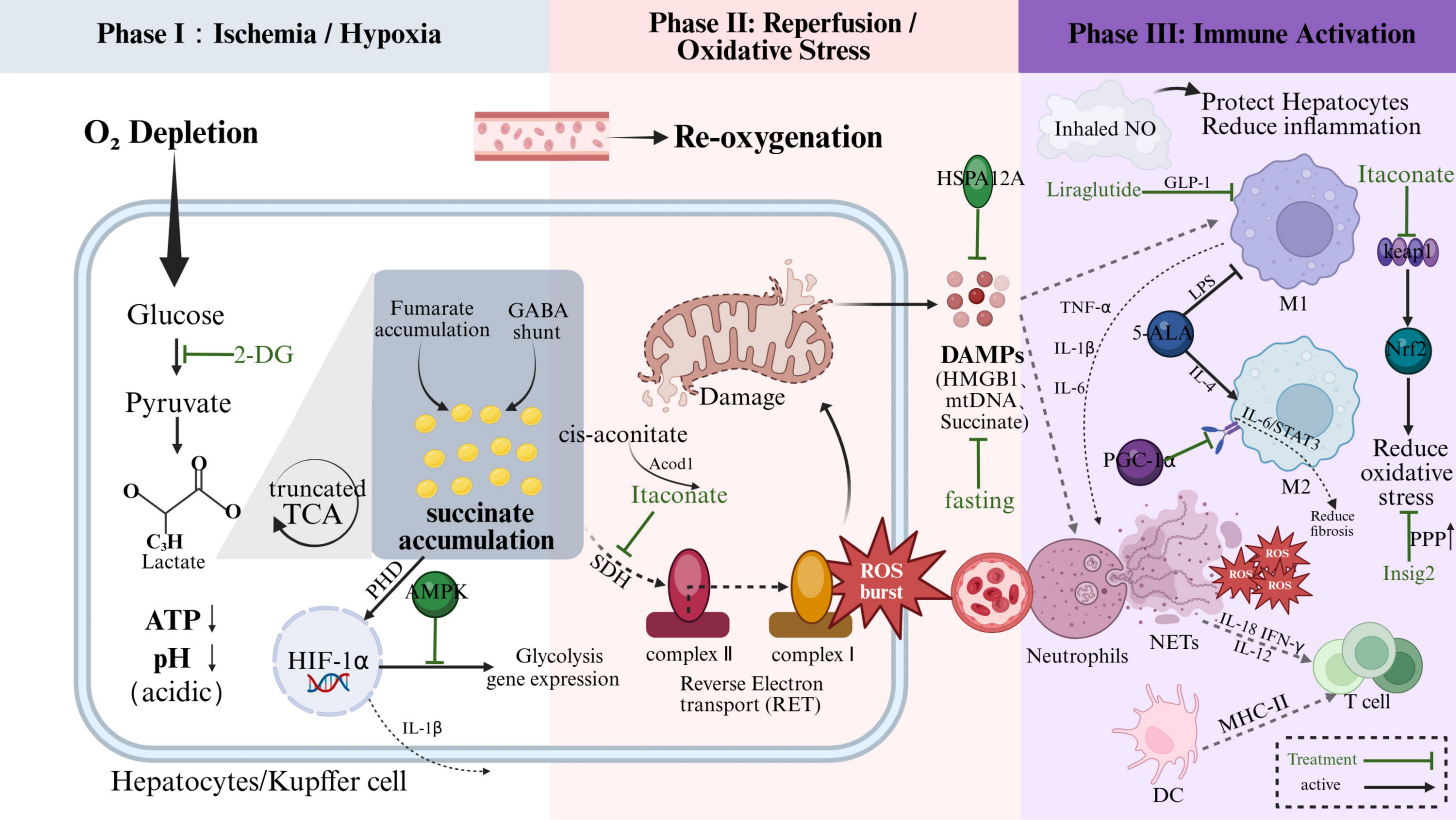
Sequential immunometabolic mechanisms and therapeutic interventions in Hepatic Ischemia-Reperfusion Injury (IRI). The pathophysiology of IRI is depicted across three distinct phases.Phase I (Ischemia/Hypoxia): Oxygen deprivation forces hepatocytes and resident Kupffer cells to shift towards anaerobic glycolysis. This results in intracellular ATP depletion, lactate accumulation, and acidosis (pH↓). Hypoxia prevents the degradation of HIF-1α, while the reversal of Succinate Dehydrogenase (SDH) activity leads to significant succinate accumulation. Phase II (Reperfusion/Oxidative Stress): Re-oxygenation triggers a massive burst of Reactive Oxygen Species (ROS) from mitochondria. This is primarily driven by the rapid oxidation of accumulated succinate via Reverse Electron Transport (RET) at mitochondrial Complex I. Phase III (Immune Activation): Oxidative stress and released Damage-Associated Molecular Patterns (DAMPs, e.g., HMGB1, mtDNA) activate neutrophils (inducing NETs formation) and recruit monocyte-derived macrophages. Therapeutic Strategies: Potential immunometabolic targets are highlighted in green. Itaconate inhibits SDH to limit succinate oxidation; Liraglutide (GLP-1 analog) and Inhaled NO exert anti-inflammatory and hepatoprotective effects; 5-ALA reduces ROS generation; and Fasting suppresses HMGB1 release. IRI, Ischemia-reperfusion injury; ROS, Reactive oxygen species; RET, Reverse electron transport; DAMPs, Damage-associated molecular patterns; NETs, Neutrophil extracellular traps; SDH, Succinate dehydrogenase; GLP-1, Glucagon-like peptide-1; 5-ALA, 5-Aminolevulinic acid; HMGB1, High mobility group box 1; Keap1-Nrf2, Kelch-like ECH-associated protein 1-Nuclear factor erythroid 2-related factor 2.

Finally, nitric oxide (NO) is a crucial effector molecule of macrophages and DCs (115). Importantly, a clinical trial demonstrated that inhaled NO accelerates liver function recovery in human liver transplant recipients, while sodium nitrite has also shown protective effects against hepatocyte apoptosis in experimental settings (116, 117) (**Figure 3**).

### 4.4 Monitoring metabolites to assess host immune balance establishment

Currently, there are no direct measures to assess the immunosuppressive state within the graft. Routine detection of post-transplant rejection typically involves monitoring drug levels and biochemical markers of liver inflammation when liver enzymes rise, relying on liver biopsy as the gold standard for diagnosing rejection. However, liver enzymes cannot distinguish between rejection, infection, or biliary obstruction, and the pharmacokinetics of drug concentrations vary significantly among individuals, making them poor indicators of immune balance. Moreover, biopsies are expensive, time-consuming, and invasive. This necessitates the search for more cost-effective, accessible, and safe non-invasive biomarkers (118).

Metabolomics research has identified several biomarkers with potential diagnostic value for evaluating allograft status. A study of 43 pediatric liver transplant recipients revealed that among 11 tryptophan-related metabolites, levels of kynurenine were significantly elevated in the acute rejection group, likely associated with IDO-mediated T cell suppression (119, 120). While the kynurenine-IDO pathway is classically recognized for driving immune tolerance, its elevation during acute rejection requires a nuanced interpretation. Rather than indicating a tolerant state, this paradoxical accumulation likely acts as a metabolomic signature of a compensatory negative feedback loop. During severe allograft inflammation, the massive release of pro-inflammatory cytokines (particularly IFN-γ) potently induces IDO expression in an attempt to suppress the ongoing T cell-mediated alloimmune attack (121). Thus, elevated kynurenine in this context serves as a stress-induced biomarker reflecting the intensity of the rejection response. Simultaneously, levels of nine key metabolites involved in bile acid synthesis were found to be elevated in the rejection group (120). Another study using LC/MS identified linoleic acid and γ-linolenic acid (associated with the COX pathway), as well as citrulline (associated with the NOS pathway), as biomarkers for rejection (122).

Immunometabolism represents an emerging strategy. Targeting the metabolic processes of immune cell activation, differentiation, and proliferation holds promise for inducing immune tolerance and reducing rejection. However, due to potential side effects and individual heterogeneity, the safety and feasibility of these novel therapies in clinical application remain to be validated. Furthermore, metabolomics technologies (e.g., mass spectrometry, NMR) are complex and lack standardized protocols for data analysis. In conclusion, elucidating the specific metabolic mechanisms of immune cells provides a novel perspective for post-transplant management. Beyond metabolites, post-transcriptional regulators (micro-RNAs), genetic biomarkers (mRNA, dd-cfDNA), and donor-specific antibodies (DSAs) also offer new options for monitoring rejection (118).

## 5 Discussion

Clinical practice in liver transplantation is currently at a pivotal transition from purely “immunosuppression” to “integrated immuno-metabolic management.” This review has systematically elucidated the central role of immune cell metabolic remodeling in transplant immunity, revealing that intracellular metabolic programs—such as the switch between glycolysis and oxidative phosphorylation—serve not only as energy supports for immune activation but also as critical checkpoints determining immune cell fate (rejection versus tolerance). This paradigm shift offers a novel theoretical framework for resolving the long-standing clinical trade-off between “allograft rejection and drug toxicity: by precisely modulating metabolic pathways, it is possible to induce specific immune tolerance while potentially mitigating metabolic syndrome and ischemia-reperfusion injury caused by broad-spectrum immunosuppressants.

However, despite the immense therapeutic potential of immunometabolic theories, translating them into mature clinical protocols remains fraught with severe dilemmas and challenges.

First, the metabolic environment of transplant recipients is characterized by high complexity and dynamism. Current research predominantly focuses on the metabolic characteristics of specific immune cell subsets in *in vitro* or static models, often overlooking the profound impact of the host’s systemic metabolic state on the local graft microenvironment. Post-operatively, liver transplant recipients undergo a drastic dynamic evolution, transitioning from the metabolic failure of end-stage liver disease and perioperative stress hypermetabolism to the potential development of metabolic syndrome during recovery. It remains unclear how systemic metabolic disturbances (e.g., hyperlipidemia, insulin resistance) cross tissue barriers to interact with the local immunometabolic network of the graft. This lack of understanding regarding the “systemic-local” metabolic crosstalk mechanism implies that intervention strategies targeting a single cellular metabolic pathway may fail to achieve anticipated efficacy within the complex *in vivo* environment.

Second, the safety profile of systemic metabolic inhibition remains the primary barrier to clinical translation. Since fundamental metabolic pathways (e.g., glycolysis, TCA cycle) are evolutionarily conserved and ubiquitous across cell types, systemic blockade inherently lacks specificity. For instance, while glycolytic inhibitors like 2-DG effectively suppress effector T cells, they pose significant risks of cardiotoxicity and central neurotoxicity (although neurons exhibit metabolic flexibility under stress, they primarily rely on glucose homeostatically and are thus highly vulnerable to severe glycolytic restriction). Beyond acute toxicity, there is a theoretical risk of ‘metabolic plasticity’, where immune cells adaptively switch to alternative fuel sources (e.g., shifting from glycolysis to glutaminolysis or fatty acid oxidation) to evade blockade, potentially rendering long-term monotherapy ineffective.

Third, the profound inter-patient metabolic heterogeneity in the real-world transplant population significantly complicates the development of standardized protocols. Unlike genetically identical laboratory mice, transplant recipients exhibit vast variability in baseline metabolic status—ranging from sarcopenic frailty to morbid obesity, metabolic dysfunction-associated steatotic liver disease (MASLD), or pre-existing diabetes. These systemic metabolic states can epigenetically ‘imprint’ immune cells, altering their metabolic set-points and sensitivity to inhibitors. For example, T cells from a diabetic recipient may exhibit a constitutively higher glycolytic drive compared to those from a metabolically healthy individual, necessitating different therapeutic dosages.

Finally, the feasibility of implementing “precision immunometabolism” is limited by diagnostic capabilities. In real-world practice, we lack validated, rapid, and non-invasive biomarkers to monitor the *in vivo* metabolic flux of immune cells. While clinicians can routinely measure serum drug trough levels (pharmacokinetics) for tacrolimus, there are no available bedside tools to quantify ‘T cell glycolytic activity’ or ‘macrophage polarization states’ (pharmacodynamics) to guide dosage adjustments. Without such dynamic monitoring systems, titrating metabolic inhibitors involves a high risk of either under-dosing or over-dosing. To provide a realistic roadmap for clinical translation, we categorized current immunometabolic targets into three tiers based on their validation status and translational feasibility (as summarized in **Table 1**):

**Table 1:** Overview of immunometabolic therapeutic targets in liver transplantation: efficacy, risks, and translational barriers.

Strategy / target	Key mechanism	Evidence level	Clinical feasibility	Major risks & barriers
mTOR Inhibitors (Sirolimus, Everolimus)	Restrict glycolysis; Promote Treg generation	Clinical (Human); Standard of Care	High (Protocol Optimization)	Dyslipidemia; New-Onset Diabetes (NODM); Impaired wound healing.
AMPK Activators (Metformin)	Enhance FAO; Inhibit mTORC1	Pre-clinical (Rodent) (Clinical Safety High)	High (Drug Repurposing)	Lactic acidosis (risk in liver failure); Gastrointestinal intolerance.
Glycolysis Inhibitors (2-DG)	Block effector T cell expansion	Pre-clinical (Rodent); Skin/Heart grafts	Low	Neurotoxicity; Impaired cardiomyocyte function; Delayed hepatocyte regeneration.
Glutamine Antagonists (DON)	Inhibit T cell proliferation	Pre-clinical (Rodent)	Low	Gastrointestinal toxicity; Pancreatic toxicity; Non-specific inhibition.
Metabolic Pre-conditioning (Diet / Exercise)	Reduce calorie and lipid overload	Pre-clinical (Rodent) & Early Clinical	Moderate	Patient compliance; Risk of malnutrition in end-stage liver disease.

1. Clinically Applied Strategies: The mTOR signaling pathway remains one of the most extensively studied immunometabolic mechanisms in human liver transplantation. Currently, targeting this axis via mTOR inhibitors (e.g., rapamycin/everolimus) represents a primary clinical strategy utilized for CNI-sparing protocols and facilitating a pro-tolerogenic environment. However, it must be acknowledged that achieving true operational tolerance remains rare in clinical practice.
2. Translatable in 3–5 Years: We posit that repurposing ‘metabolic safety-proven’ drugs represents the most realistic avenue for the immediate future. Agents like Metformin (AMPK activator) and GLP-1 agonists (e.g., Liraglutide), which have robust safety profiles in diabetic patients, are primed for Phase II clinical trials to evaluate their efficacy in modulating post-transplant rejection and metabolic syndrome.
3. Experimental & Speculative: Conversely, direct inhibitors of primary metabolic enzymes—such as 2-DG (glycolysis), DON (glutaminolysis), and itaconate derivatives—remain largely rodent-based and speculative. Despite their efficacy in murine models, their translation is currently hindered by the risk of severe systemic toxicity and the lack of tissue-specific delivery systems.

Based on these limitations, future research and clinical translation should focus on the following directions: Utilizing single-cell sequencing and spatial metabolomics to map the dynamic immunometabolic landscape of human liver transplant recipients across different immune states (tolerance, rejection, infection). The goal is to precisely identify “metabolic-immune” dual targets that are specifically highly expressed in pathogenic immune cells but low in normal tissues, thereby minimizing therapeutic side effects. Conducting prospective clinical studies to evaluate the feasibility of existing metabolic modulators (e.g., metformin, SGLT2 inhibitors, AMPK agonists) as adjunctive immunosuppressive regimens, exploring their synergistic effects in CNI minimization, IRI mitigation, and tolerance induction. Establishing a non-invasive monitoring system based on metabolomics. Given the invasive nature and lag time of liver biopsies, developing specific metabolic biomarkers (e.g., specific lipid profiles, amino acid signatures, or exosomal contents) is of significant clinical value for early warning of rejection and dynamic adjustment of individualized immune protocols.

In conclusion, immunometabolic remodeling is not merely a mechanistic supplement to basic immunology but a critical factor in optimizing long-term outcomes in liver transplantation. Objectively assessing current translational dilemmas and establishing rigorous validation frameworks to link mechanistic elucidation with clinical application will be essential for transitioning the field from “generalized immunosuppression” toward “precision immunometabolic therapy.”
