# Correction: Immunometabolic remodeling: new perspectives and strategies for liver transplantation

**DOI:** 10.3389/fimmu.2026.1847484

**Published:** 2026-04-20

**Authors:** Longbo Wang, Xiyang Sheng, Gengyuan Shi, Yongzhao Li, Dongdong Wang, Wei Wang, Chen Mi, Siyang Wang, Yongyue Du, Hanteng Yang

**Affiliations:** Department of General Surgery, Lanzhou University Second Hospital, Lanzhou, Gansu, China

**Keywords:** immune tolerance, immunometabolism, ischemia-reperfusion injury, liver transplantation, metabolic remodeling, post-transplant metabolic syndrome

There was an error in the placement of **Figure 1**, **Figure 2**, and **Figure 3** as published. Due to a production error, the images for these figures were incorrectly sequenced: the image intended for **Figure 3** was placed as **Figure 1**, the image for **Figure 2** was placed as **Figure 3**, and the image for **Figure 1** was placed as **Figure 2**. The figure legends, however, were correctly positioned in the original article.

**Figure 1:**
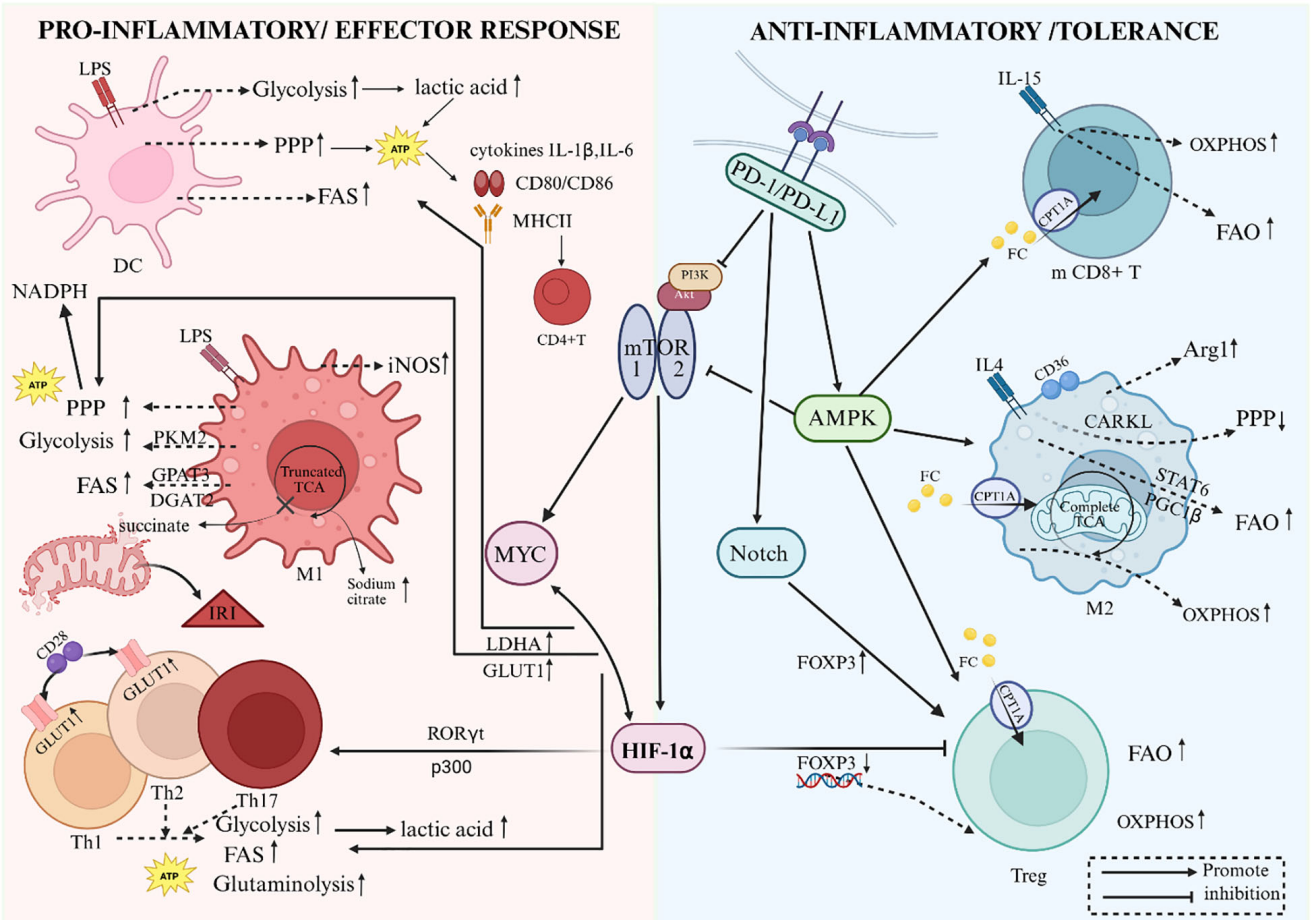
Metabolic reprogramming orchestrates immune cell differentiation and functional plasticity. The schematic illustrates the divergent metabolic programs characterizing pro-inflammatory effector cells (Left Panel) versus anti-inflammatory regulatory cells (Right Panel). (Left) Upon activation by pathogen-associated molecular patterns (e.g., LPS), Dendritic Cells (DCs) and M1 Macrophages undergo a “glycolytic switch” (Warburg effect). This process involves upregulation of the Pentose Phosphate Pathway (PPP) to generate NADPH and ATP, supporting the synthesis of pro-inflammatory cytokines (IL-1β, IL-6) and costimulatory molecules (CD80/CD86). A characteristic “truncated” TCA cycle in M1 macrophages leads to the accumulation of citrate (fueling Fatty Acid Synthesis, FAS, and NO production) and succinate. Succinate accumulation stabilizes HIF-1α, further driving glycolytic gene expression. Effector T cell subsets (Th1, Th2, Th17) similarly rely on aerobic glycolysis, glutaminolysis, and FAS, processes driven by the PI3K-Akt-mTOR-Myc signaling axis. (Right) In contrast, cells responsible for immune tolerance and resolution (M2 Macrophages, Tregs, and Memory CD8+ T cells) exhibit a metabolic preference for Oxidative Phosphorylation (OXPHOS) and Fatty Acid Oxidation (FAO). This metabolic profile is sustained by AMPK activation, which antagonizes mTOR signaling and promotes mitochondrial biogenesis via PGC-1α/b and STAT6 pathways. The PD-1/PD-L1 immune checkpoint reinforces tolerance by inhibiting glycolysis and promoting lipolysis and FAO. DC, Dendritic cell; LPS, Lipopolysaccharide; PPP, Pentose phosphate pathway; FAS, Fatty acid synthesis; FAO, Fatty acid oxidation; OXPHOS, Oxidative phosphorylation; TCA, Tricarboxylic acid cycle; HIF-1α, Hypoxia-inducible factor-1α; mTOR, Mammalian target of rapamycin; AMPK, AMP-activated protein kinase; PKM2, Pyruvate kinase M2; iNOS, Inducible nitric oxide synthase; Arg1, Arginase-1; CPT1A, Carnitine palmitoyltransferase 1A; RORγt, RAR-related orphan receptor γt.

**Figure 2:**
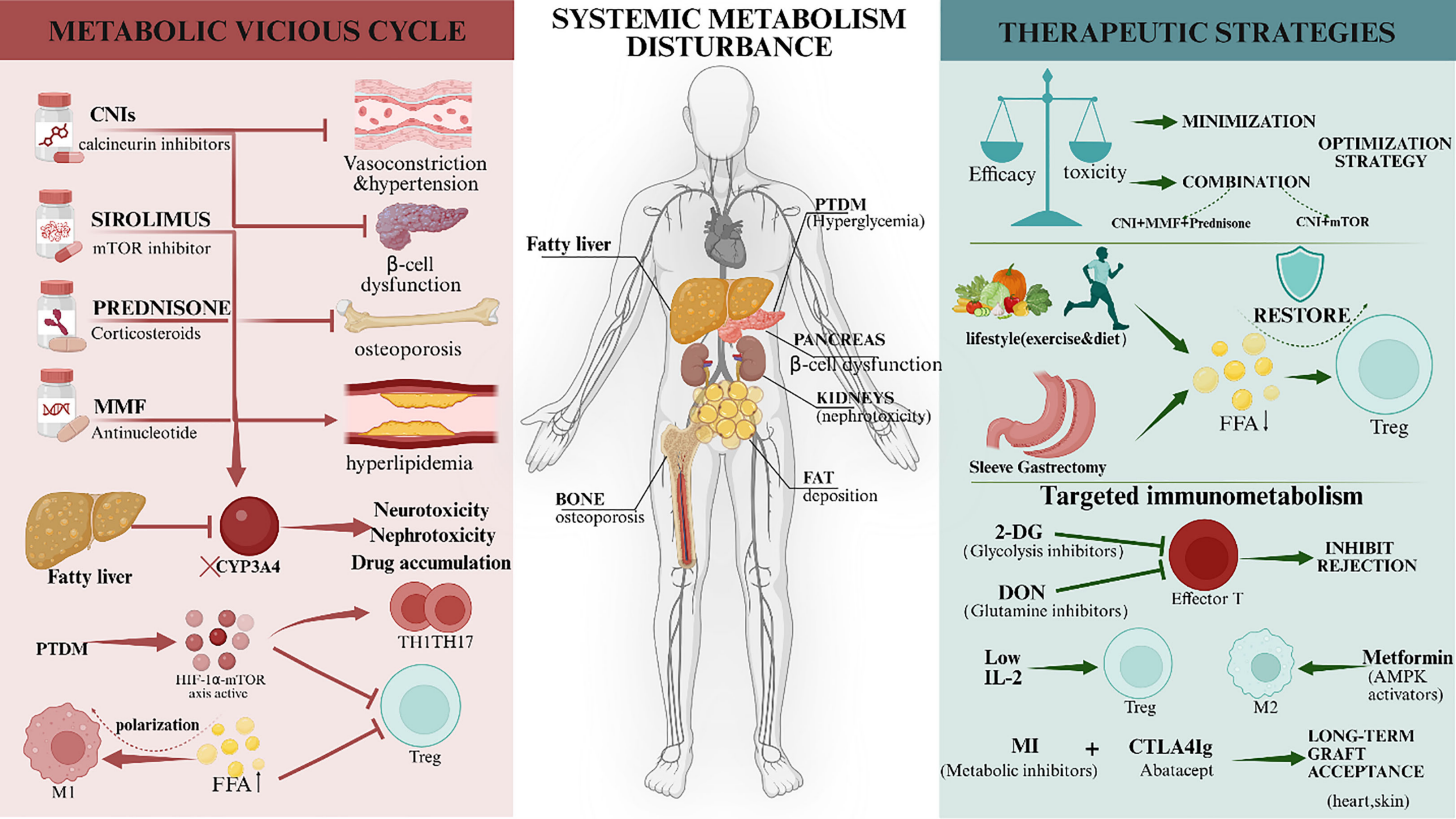
The interplay between immunosuppression-induced metabolic disorders and targeted therapeutic strategies. (Left Panel) The Metabolic Vicious Cycle: Standard immunosuppressive regimens (CNIs, mTOR inhibitors, Corticosteroids) are associated with systemic metabolic adverse events, including hypertension, b-cell dysfunction, osteoporosis, and dyslipidemia. Conversely, pre-existing host metabolic disorders (e.g., Fatty Liver/MASLD) downregulate hepatic CYP3A4 activity, leading to drug accumulation and enhanced toxicity. Systemic metabolic disturbances, such as elevated Free Fatty Acids (FFA) and hyperglycemia, promote a pro-inflammatory microenvironment by driving Th1/Th17 polarization via the mTOR/HIF-1α axis while suppressing Treg differentiation. (Right Panel) Therapeutic Strategies: Clinical management involves balancing efficacy and toxicity through minimization strategies or combination therapies (e.g., reduced CNI + mTOR inhibitors). Lifestyle interventions (Diet, Exercise) and Bariatric Surgery (Sleeve Gastrectomy) help restore metabolic homeostasis. Emerging “Targeted Immunometabolism” approaches aim to induce graft tolerance using specific metabolic modulators: 2-DG (Glycolysis inhibitor) and DON (Glutamine inhibitor) to suppress effector T cells; Metformin to activate AMPK and enhance M2/Treg function; and synergistic combinations with CTLA4-Ig to promote long-term graft acceptance. CNI, Calcineurin inhibitors; MMF, Mycophenolate mofetil; PTDM, Post-transplant diabetes mellitus; FFA, Free fatty acids; CYP3A4, Cytochrome P450 3A4; 2-DG, 2-Deoxy-D-glucose; DON, 6-Diazo-5-oxo-L-norleucine.

**Figure 3:**
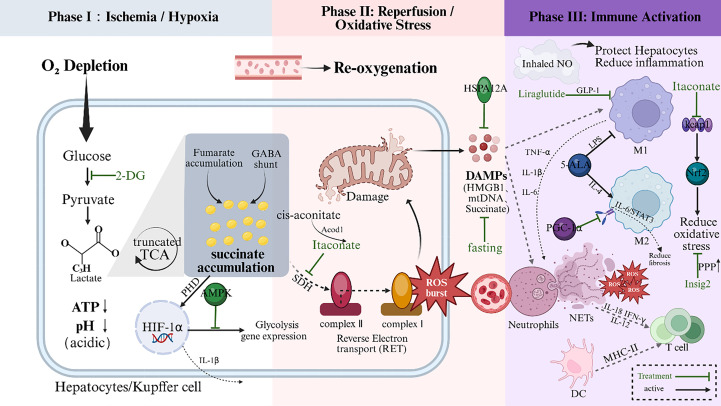
Sequential immunometabolic mechanisms and therapeutic interventions in Hepatic Ischemia-Reperfusion Injury (IRI). The pathophysiology of IRI is depicted across three distinct phases.Phase I (Ischemia/Hypoxia): Oxygen deprivation forces hepatocytes and resident Kupffer cells to shift towards anaerobic glycolysis. This results in intracellular ATP depletion, lactate accumulation, and acidosis (pH↓). Hypoxia prevents the degradation of HIF-1α, while the reversal of Succinate Dehydrogenase (SDH) activity leads to significant succinate accumulation. Phase II (Reperfusion/Oxidative Stress): Re-oxygenation triggers a massive burst of Reactive Oxygen Species (ROS) from mitochondria. This is primarily driven by the rapid oxidation of accumulated succinate via Reverse Electron Transport (RET) at mitochondrial Complex I. Phase III (Immune Activation): Oxidative stress and released Damage-Associated Molecular Patterns (DAMPs, e.g., HMGB1, mtDNA) activate neutrophils (inducing NETs formation) and recruit monocyte-derived macrophages. Therapeutic Strategies: Potential immunometabolic targets are highlighted in green. Itaconate inhibits SDH to limit succinate oxidation; Liraglutide (GLP-1 analog) and Inhaled NO exert anti-inflammatory and hepatoprotective effects; 5-ALA reduces ROS generation; and Fasting suppresses HMGB1 release. IRI, Ischemia-reperfusion injury; ROS, Reactive oxygen species; RET, Reverse electron transport; DAMPs, Damage-associated molecular patterns; NETs, Neutrophil extracellular traps; SDH, Succinate dehydrogenase; GLP-1, Glucagon-like peptide-1; 5-ALA, 5-Aminolevulinic acid; HMGB1, High mobility group box 1; Keap1-Nrf2, Kelch-like ECH-associated protein 1-Nuclear factor erythroid 2-related factor 2.

The correct **Figure 1**, **Figure 2**, and **Figure 3** appear below.

The original version of this article has been updated.

